# Characterization and Biotechnological Potential of Extracellular Polysaccharides Synthesized by *Alteromonas* Strains Isolated from French Polynesia Marine Environments

**DOI:** 10.3390/md19090522

**Published:** 2021-09-17

**Authors:** Patrícia Concórdio-Reis, Vítor D. Alves, Xavier Moppert, Jean Guézennec, Filomena Freitas, Maria A. M. Reis

**Affiliations:** 1 Associate Laboratory i4HB–Institute for Health and Bioeconomy, School of Science and Technology, NOVA University Lisbon, 2829-516 Caparica, Portugal; pc.reis@campus.fct.unl.pt (P.C.-R.); amr@fct.unl.pt (M.A.M.R.); 2 UCIBIO–Applied Molecular Biosciences Unit, Department of Chemistry, School of Science and Technology, NOVA University Lisbon, 2829-516 Caparica, Portugal; 3 LEAF—Linking Landscape, Environment, Agriculture and Food—Research Center, Instituto Superior de Agronomia, Universidade de Lisboa, 1349-017 Lisbon, Portugal; vitoralves@isa.ulisboa.pt; 4 Pacific Biotech SAS, BP 140 289, 98 701 Arue, Tahiti, French Polynesia; xmoppert@pacific-biotech.pf; 5 AiMB (Advices in Marine Biotechnology), 17 Rue d’Ouessant, 29280 Plouzané, France; guezennec.jean@wanadoo.fr

**Keywords:** *Alteromonas* sp., exopolysaccharide characterization, functional properties, rheology, gelation

## Abstract

Marine environments comprise almost three quarters of Earth’s surface, representing the largest ecosystem of our planet. The vast ecological and metabolic diversity found in marine microorganisms suggest that these marine resources have a huge potential as sources of novel commercially appealing biomolecules, such as exopolysaccharides (EPS). Six *Alteromonas* strains from different marine environments in French Polynesia atolls were selected for EPS extraction. All the EPS were heteropolysaccharides composed of different monomers, including neutral monosaccharides (glucose, galactose, and mannose, rhamnose and fucose), and uronic acids (glucuronic acid and galacturonic acid), which accounted for up to 45.5 mol% of the EPS compositions. Non-carbohydrate substituents, such as acetyl (0.5–2.1 wt%), pyruvyl (0.2–4.9 wt%), succinyl (1–1.8 wt%), and sulfate (1.98–3.43 wt%); and few peptides (1.72–6.77 wt%) were also detected. Thermal analysis demonstrated that the EPS had a degradation temperature above 260 °C, and high char yields (32–53%). Studies on EPS functional properties revealed that they produce viscous aqueous solutions with a shear thinning behavior and could form strong gels in two distinct ways: by the addition of Fe^2+^, or in the presence of Mg^2+^, Cu^2+^, or Ca^2+^ under alkaline conditions. Thus, these EPS could be versatile materials for different applications.

## 1. Introduction

Sea water covers more than 70% of the earth’s surface and represents the largest ecosystem of the planet. Despite the enormous metabolic diversity and discovery potential, marine natural resources are still underexplored, understudied, and underutilized in biotechnology [1,2]. From the shallow coastal waters to the deep ocean, marine habitats have a variety of unique ecological characteristics that prompted marine microbial communities to develop adaptation mechanisms in order to survive [2,3]. This is especially true in extreme environments such as those found in deep-sea, hydrothermal vents, volcanic and hydrothermal marine areas, marine salterns, and sea ice in polar regions [4].

Important molecules for cell survival are extracellular polysaccharides (EPS), high molecular weight carbohydrate polymers secreted by many microorganisms, particularly bacteria [5,6]. Bacterial EPS make up a substantial part of the extracellular polymers that surround cells in microbial communities [7]. A variety of functions have been attributed to EPS, including adhesion to surfaces, cell–cell recognition and aggregation, entrapment of nutrients, and protective barrier [8,9,10]. EPS are especially important for marine bacteria to thrive under the extreme conditions characteristic of their environment, such as high pressure, high salinity, low nutrient concentration, low or high temperature, and heavy metal presence [11]. Moreover, bacterially synthesized EPS are commercially appealing due to their interesting physicochemical and functional properties, with potential applicability as biomaterials, rheology modifiers of aqueous solutions, or bioactive therapeutic agents [10,12]. In fact, marine bacteria are currently a very promising source for the discovery of EPS with distinctive structures and unique properties, with applicability in several industrial sectors [1,13]. These EPS often exhibit a high diversity in structure and composition, that might include rare sugars, such as fucose or rhamnose [13,14], which are known to confer the biopolymers’ biological activity [12]. High content in uronic acids and sulfate groups are also found within marine bacteria derived EPS, which further contributes to their unique properties [13].

In the course of discovery of biomolecules of biotechnological interest, it is well accepted that microorganisms originating from unusual environments, such as deep-sea hydrothermal vents, Antarctica, and microbial mats located in some Polynesian atolls, will provide a valuable source for novel biopolymers, including EPS with unique properties [1,15]. Microbial mats (locally called “Kopara” mats) are characterized by salt concentrations ranging from 5 to 42 g L^−1^, pH values between 6 to 10.5, and temperatures ranging from 20 °C during the night, up to 42 °C [16]. To date, only five genera of EPS-producing bacteria were found in such environments, i.e., *Pseudomonas*, *Pseudoalteromonas*, *Alteromonas*, *Paracoccus*, and *Vibrio*, with uronic acids and amino-sugars content up to 50% (*w*/*w*), as well as acyl and sulfate groups, in their secreted EPS composition, which render them unique properties and great potential for use in several applications, including in the cosmetic, biomedicine, pharmaceutical, and therapeutics fields [16].

In this study, six *Alteromonas* strains originating from different locations in French Polynesia were screened for EPS production in an aerobic, carbohydrate-based medium. The resulting EPS were characterized in terms of chemical composition, molecular mass distribution, and thermal properties. To investigate their potential as biomaterials in important biotechnological applications, the functional properties of the biopolymers were assessed, including determination of their rheological properties and gel forming capacity.

## 2. Results and Discussion

### 2.1. Isolation and Identification of EPS Producing Strains

**Figure 1:**
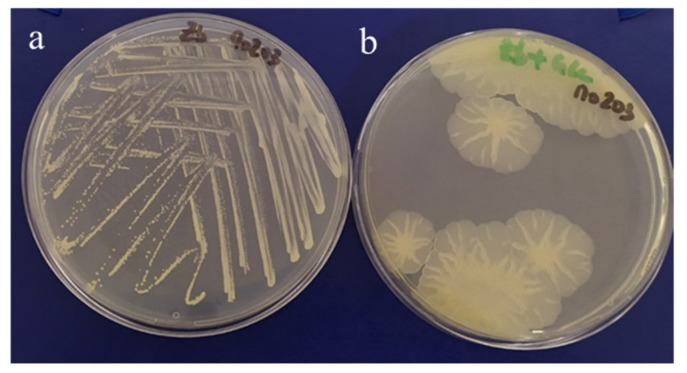
Culture of strain Mo 203 on Zobell-agar medium (**a**) and glucose-supplemented (30 g L^−1^) Zobell-agar medium (**b**).

The six *Alteromonas* isolates used in this study were selected based on their ability to show a mucoid phenotype (Figure 1). Strains Mo 169, Mo 278, and Mo 203 were originally isolated from a giant clam, a sea anemone, and a “Kopara” mat sample, respectively, collected at Moorea Island lagoon (Society Archipelago). Strains Fak 1576 and Fak 1386 were isolated from a coral and a pearl oyster mantle, respectively, at the Fakarava atoll, while strain Tik 650 was isolated from a “Kopara” mat sample collected at Tikehau atoll (Table 1). Upon taxonomical analysis, the strains were identified as belonging to the *Alteromonas macleodii* specie, except for strain Tik 650 identified as a *Alteromonas simiduii*.

### 2.2. EPS Characterization

#### 2.2.1. Chemical Composition

The *Alteromonas* EPS were heteropolysaccharides composed of different monomers, including neutral monosaccharides (glucose, galactose, and mannose, rhamnose, and fucose) and uronic acids (glucuronic acid and galacturonic acid) (Table 2).

Almost all biopolymers were rich EPS in uronic acids, with glucuronic acid (GlcA) and galacturonic acid (GalA) accounting for up to 45.5 mol%. Such high contents in uronic acids were also reported for EPS produced by deep-sea hydrothermal bacterium *A. infernus* GY 785 [17], and *Alteromonas* sp. JL2810 collected from surface seawater [11]. Due to the uronic acids in their composition, both *Alteromonas* strains showed a high metal binding capacity, leading to a possible use in wastewater treatment and metal recovery [11,18]. On the other hand, no uronic sugars were detected in the EPS produced by *A. hispanica* F32 isolated from a hypersaline environment [19] and two strains of *A. stellipolaris*, namely, strains PQQ-42 and PQQ-44, isolated from a fish hatchery [20].

The rare sugar rhamnose (Rha) was also present in significant proportions in EPS C, E, and F. Although rhamnose was previously reported in EPS from *Alteromonas* strains [11,17,19,20,21], only a few exhibited significant amounts of this sugar in their composition. Examples include the EPS produced by *A. macleodii* subsp. *fijiensis* biovar deepsane HYD 657 [21], and the EPS from *Alteromonas* sp. JL2810 that consisted in a trisaccharide repeating unit composed of Rha, GalA, and mannose (Man) [11]. EPS C also contained fucose (Fuc) in its composition, although at low quantities. The presence of rare sugars, such as Rha and Fuc, or uronic acids makes these EPS excellent candidates for various applications, such as anti-inflammatory and antioxidant agents, or in the synthesis of nucleoside analogs used as antiviral substances [22]. Moreover, these EPS can be used as sources of rare sugar monosaccharides with high value applications. For instance, rhamnose is used as precursor in the synthesis of aroma and flavors [22], whereas fucose is often present in the composition of anticarcinogenic and anti-inflammatory drugs, and incorporated in skin care formulations for the acceleration of wound healing [22].

Non-sugar substituents, such as acetyl, pyruvyl, succinyl, and sulfate, were determined in the *Alteromonas* EPS (Table 2). All the six EPS had at least one type of acyl substituent with a total acyl content accounting for 1 to 5.5% of the EPS dry mass. EPS A and F were particularly rich in pyruvyl groups (4.9–5.5 wt%) while EPS E and B exhibited a higher content in succinyl substituents (1 and 1.8 wt%, respectively). EPS D only had acetyl groups, which accounted for 2.1 wt% of the EPS’ mass. Acetyl and pyruvyl substituents, although rarely, have been reported in a few EPS produced by *Alteromonas* strains [19,21]. On the other hand, succinyl, to the best of our knowledge, has never been found in *Alteromonas* EPS. Additionally, sulfate substituents were found in all EPS, but in low concentrations, ranging from 2.0–3.4 wt% (Table 2). Although more commonly found in algae, sulfated EPS have been described to be produced by some marine bacteria [2], including *A. hispanica* F32 (0.25 wt%) [19], *A. infernus* GY785 (3 wt%) [17], and *Alteromonas* HYD 657 (7.5 wt%) [21]. Sulfated polysaccharides are of great interest in the fields of biomedicine, pharmaceuticals, nutraceutical foods, and cosmetics due to their biological activity [2]. Reported bioactive properties include anti-inflammatory, immunomodulatory, antioxidant, antiviral, antibacterial, antiulcer, antitumor and antihyperlipidaemic activity, as well as anti-coagulant and/or anti-thrombotic properties [23,24,25,26]. In fact, Sahana and Rekha [13] recently reported that the EPS produced by *Alteromonas* HYD 657 was biocompatible, capable of promoting cell proliferation, and contributed to wound healing in vitro.

After the purification process, the EPS samples presented some content of protein and inorganic salts (Table 2). The presence of a residual protein content (1.7–6.8 wt%) in the EPS was previously reported for EPS produced by other *Alteromonas* strains (0–8 wt%) [13,19,21,27]. The inorganic salts content of EPS B, C, and E (15.0–34.7 wt%) was similar to those reported (15.5–40 wt%) for *A. hispanica* F32 [19] and *Alteromonas* sp. PRIM-28 [13], whereas EPS A, D, and F had a comparatively lower content (7.7–13.7 wt%).

#### 2.2.2. Fourier Transform Infra-Red (FTIR) Spectroscopy

**Figure 2:**
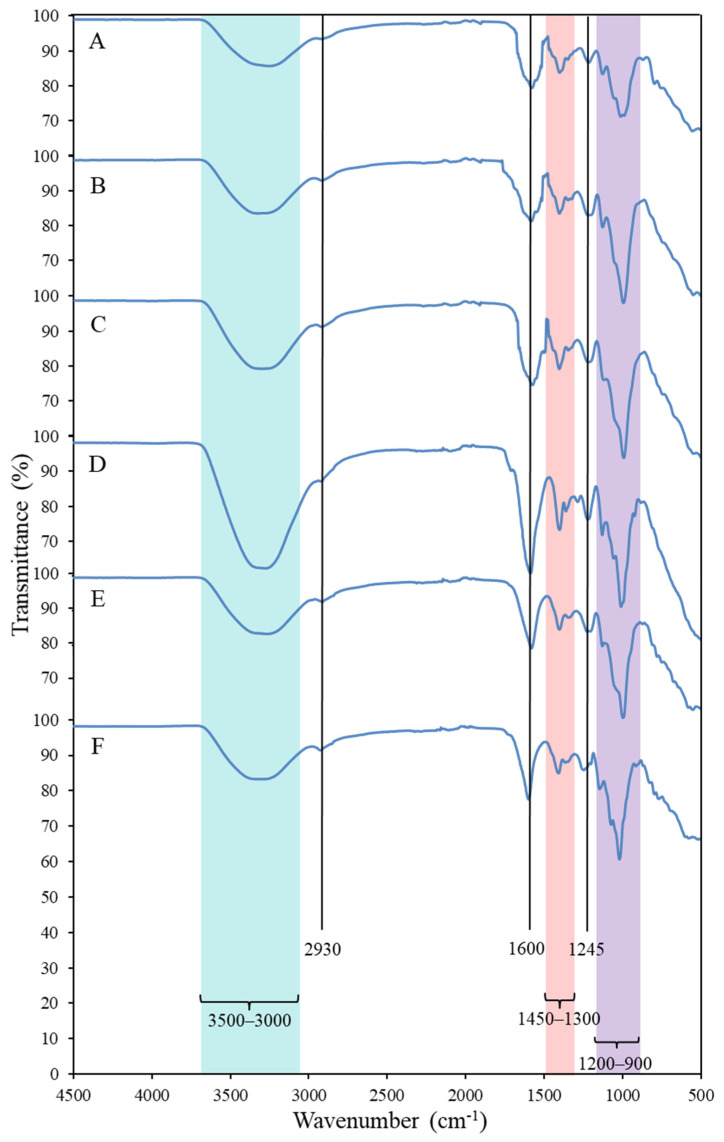
FTIR spectra of EPS A–F produced by *Alteromonas* strains isolated from French Polynesia.

The FTIR spectra of the EPS, presented in Figure 2, contained bands typically found in carbohydrates. The broad and intense band observed in the frequency range of 3500–3000 cm^−1^ corresponded to the O-H stretching vibration of hydroxyl groups, which partially overlaps the C-H stretching peak of CH_2_ groups that appeared at around 2930 cm^−1^ [28,29]. The absorption peak at approximately 1600 cm^−1^ and the adsorption region between 1300 and 1450 cm^−1^ are characteristic of the C=O asymmetric and symmetric stretching vibrations, respectively, of the carboxylates from the uronic acids [28,29,30]. The band found at around 1245 cm^−1^ can be attributed to the stretching vibration of C-O-C from acyls, and/or of S=O from sulphate groups [28,30,31]. Finally, the bands in the frequency range of 1200–900 cm^−1^ can be assigned to C-O and C-C vibrations of the glycosidic bonds and pyranose carbohydrate ring [28,30].

#### 2.2.3. Molecular Mass Distribution

**Figure A1:**
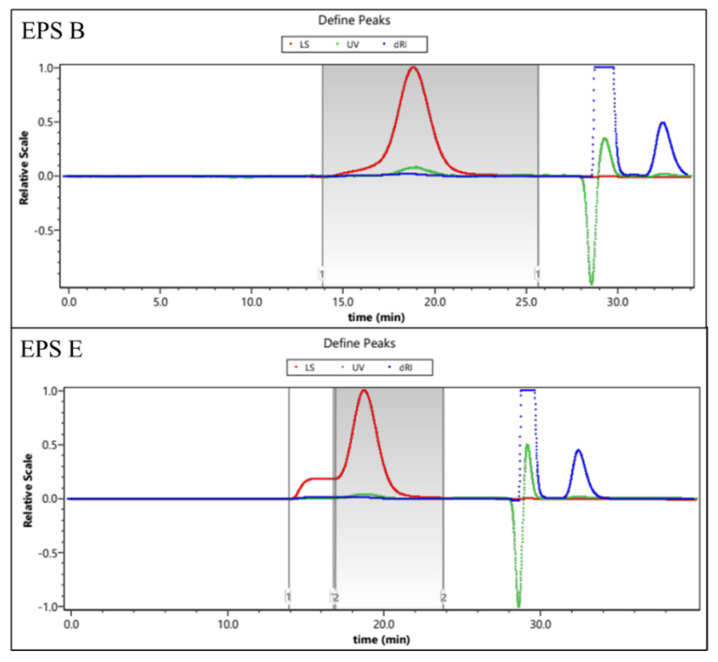
Molecular weight determination of EPS B and EPS E.

EPS characterization in terms of composition and average molecular weight is presented in Table 2. Regarding the average molecular weights (M_w_), all the EPS produced by *Alteromonas* strains presented a high Mw value, ranging from 1.2–4.6 MDa (Table 2). These Mw values are within the ranges reported for commercial EPS, such as xanthan, alginate, and hyaluronic acid (0.3–50 MDa) [12], and for other *Alteromonas* EPS (1.1–2 MDa), such as those produced by *Alteromonas infernus* GY785 [17], *A. macleodii* subsp. *fijiensis* HYD 657 [21], and *A. macleodii* subsp. *fijiensis* biovar *medioatlantica* [27]. However, these values were higher than the Mw estimated for the EPS obtained from *Alteromonas* sp. PRIM-28 [13] and *Alteromonas* sp. JL2810 [11], which were 780 kDa and 0.17 MDa, respectively, and lower than 19.7 MDa, the highest Mw reported so far for *Alteromonas* EPS [19]. Interestingly, two distinct chromatographic peaks were detected in EPS A and EPS E, suggesting that strains Mo 169 and Fak 1386 might synthesize two types of high molecular weight EPS. Examples of the two distinctive HPSEC results, one and two chromatographic peaks, are presented in Appendix A (Figure A1). All EPS presented low PDI values, thus indicating their homogeneity in terms of molecules chain length (Table 2).

#### 2.2.4. Thermogravimetric Analysis

**Figure 3:**
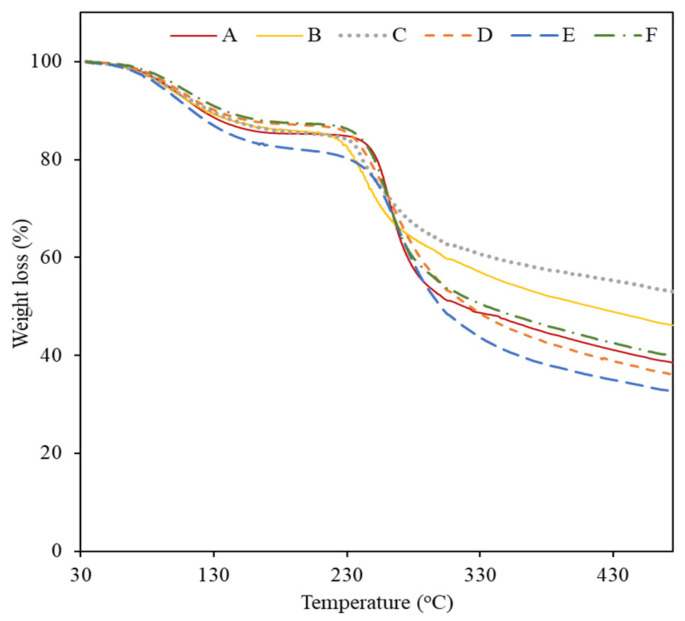
Thermogravimetric analysis (TGA) curves of EPS produced by *Alteromonas* strains isolated from French Polynesia.

The applicability of materials is also dependent on their thermal characteristics; thus, the thermal stability of the six EPS was studied using thermogravimetric analysis (TGA) (Figure 3). The thermal degradation of all the EPS occurred in three phases. In the first phase, an increase in temperature from 37–45 °C to 173–193 °C resulted in a decrease in weight of 12.1–17.5% (degradation steps are presented in Appendix B, Table A1), which was probably related to moisture loss [28,32]. The highest mass loss value (17.5%) was observed for EPS E, which suggests a strong water binding capacity. Above this temperature, a significant weight loss of 30.2–45.1% occurred until 386–429 °C (Figure 3 and Table A1), which corresponded to the second degradation phase, probably related to the decomposition of the polysaccharide’s side chains [28]. The EPS’s thermal degradation temperature (T_deg_) was similar in all EPS, varying between 260 °C and 269 °C (Table 2). Finally, as temperatures rose above 386–429 °C, a gradual decrease in mass was observed for all EPS (2.5–6.4%), identified as the third step of thermal degradation (Figure 3), which is associated with polysaccharide’s main-chain scission [28]. All the EPS showed high char yields (32–53%), with EPS C and B presenting the highest results (53% and 46%, respectively). This trend is in agreement with previous results, as both EPS also presented the highest inorganic content in their composition (Table 2).

### 2.3. EPS Functional Properties

Polysaccharides are excellent candidates for biomaterials with applications in several industries due to their valuable rheological properties, as well as the ability to form polymeric structures, such as films, gels, and emulsions [22].

#### 2.3.1. Rheological Properties

**Figure 4:**
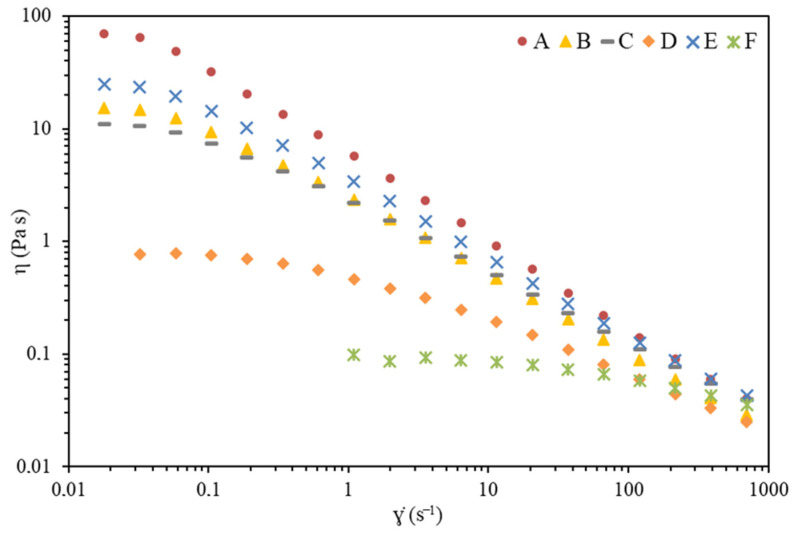
Apparent viscosity (η) as a function of shear rate ($\dot{ɣ}$) for different aqueous solutions (1% *w*/*w*) prepared with EPS produced by *Alteromonas* strains isolated from French Polynesia.

The flow curves obtained for purified EPS solutions (1 wt%) are presented in Figure 4. It can be observed that the EPS have diverse thickening capacities, as they show different values of apparent viscosity for the same shear rate, with the following order: A > E > B~C > D > F. This trend is clearly visible at the lower shear rate values tested, where the curves present or approach the first Newtonian plateau at which a high degree of polymer chains interactions (e.g., entanglements) is expected, and the molecules show high flow resistance and thereby higher viscosity [33,34].

The Newtonian plateau at low shear rates followed by a shear thinning behavior observed for all *Alteromonas* sp. EPS solutions is in line to that reported for other polysaccharide aqueous solutions [33,34,35,36,37]. This viscosity dependence with the shear rate is commonly described by mathematical models, such as the Carreau model [38]:

(1) $$\eta =\eta_{\infty }+\frac{\eta_{0}+\eta_{\infty }}{{\left[1+{\left(\lambda \;\dot{\gamma }\right)}^{2}\right]}^{\frac{1-n}{2}}}$$

where *η* is the apparent viscosity (Pa.s), $\dot{ɣ}$ is the shear rate (s^−1^), *λ* is a time constant (s), *η*_0_ (Pa.s) is the zero-shear rate viscosity (first Newtonian plateau), $\eta_{\infty }$ is the viscosity of the second Newtonian plateau (Pa.s), and *n* is the viscosity exponent. Equation (1) was simplified assuming $\eta_{\infty }$ values much lower than *η*_0_ and *η*, fitted to the data of Figure 4 and the estimated parameter values are presented in Table 3.

EPS A shows a higher thickening effect, with an estimated *η*_0_ of 76.2 ± 2.1 Pa.s, followed by a quick transition to a strong shear thinning illustrated by the low viscosity exponent (*n* = 0.181 ± 0.051). This behavior can be compared with that demonstrated by 1 wt.% xanthan solution [37]. As the viscosity decreases, by the order indicated above, the time constant values also decrease. As the time constant is a relaxation time, it means that, under lower viscosity values, the rate of formation of new interactions between molecules increases over the rate of their disruption. Consequently, the transition from Newtonian plateau to shear-thinning regimes moves to higher shear rate values, as observed (Figure 4) [35,36]. Polysaccharides with shear thinning behavior are commercially relevant in specific areas such as cosmetics, food products, pharmaceuticals, oil drilling fluids, and paints [39].

An evident correlation is not detected between the viscosity properties and the average molecular weight and chemical composition of the EPS presented in Table 2. The interactions between polymer chains in solutions are strongly dependent, not only on such factors, but also on the on the polymers chemical structure. As such, deeper studies are needed to evaluate the effect of single factors (e.g., chemical composition and structure, average molecular weight, charge density, polymer concentration, temperature, and ionic strength) and their interaction on the observed apparent viscosity.

#### 2.3.2. Gel Forming Capacity

**Figure 5:**
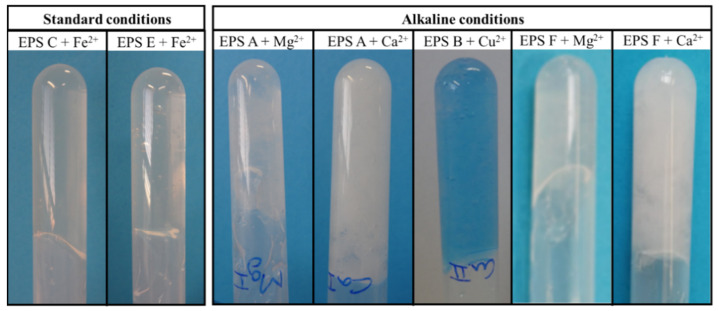
Results from gel formations type (+) with different cations, under standard and alkaline conditions.

**Figure A2:**
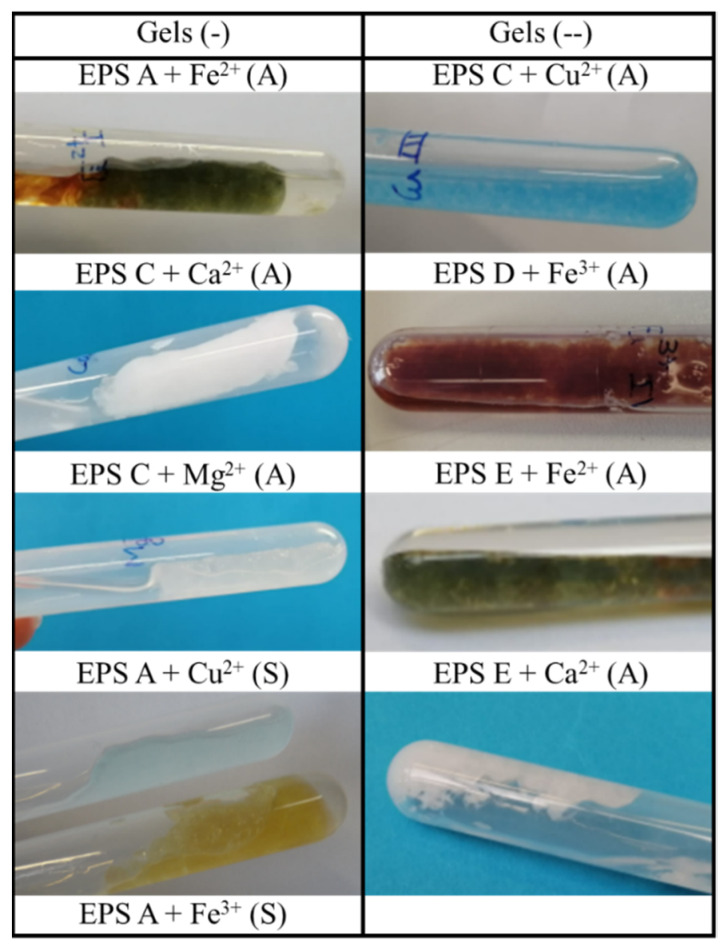
Results from gel formations type (-) and (--) with different cations under standard and alkaline conditions.

The presence of negatively charged groups (uronic acids, sulfate, and acyl substituents) render EPS a polyelectrolyte character that allows the formation of physical cross-linking interactions with cations, leading to gel formation [2,40]. The EPS gelation ability was tested using different polyvalent cations (Mg^2+^, Cu^2+^, Ca^2+^; Fe^2+^, and Fe^3+^), under standard and alkaline conditions (Table 4). Gel formation was assessed according with their strength and homogeneity: (+) for homogeneous gels that maintained their gel structure in an inversion test (Figure 5), (-) for homogeneous gels that did not sustain their structure in the inversion test, and (--) for small non-homogeneous gels. Images of gels (-) and (--) are presented in Appendix C (Figure A2). As shown in Table 4, under standard conditions, only EPS C and E were able to form strong homogenous gels with Fe^2+^ (Figure 5). Interestingly, both EPS were majorly composed of galactose (Gal) and, with the exception of Fuc in EPS C, presented the same sugar monomers but in different ratios (Table 2). Moreover, both had the lowest uronic acid content (around 17 and 22 mol%, respectively) of the six EPS tested. Cu^2+^ and Fe^3+^ were capable of promoting gelation of EPS A and C; and A and D, respectively. Although homogenous, these gels were weaker, and did not maintain their structure upon inversion.

Overall, alkaline conditions seemed to improve gelation as five combinations originated strong gels (Table 4, Figure 5). For EPS A and F, strong gelation occurred in the presence of Mg^2+^ and Ca^2+^, and a weak gel was formed in the presence of Fe^2+^. In terms of their compositions, EPS A and F had high uronic acid content (42.1 and 37.5 mol%, respectively) and similar acyl compositions, namely both were rich in pyruvate and had acetate in smaller amounts (Table 2). Moreover, EPS B formed a strong gel only with Cu^2+^ cations (Table 4, Figure 5). EPS B is composed of Gal, GlcA, Glc, and Gal A and, compared with the other polymers, had the highest uronic acid content (45.5 mol%) (Table 2). Similarly, the two *Vibrio* EPS, HE800, and MO245, formed strong gels with Cu^2+^, but not with other cations, such as Ca^2+^ [4,41]. The authors suggested that these results were due to the stronger affinity of uronic acid rich polysaccharides for Cu^2+^ compared to Ca^2+^ [4], which is in accordance with our findings. The presence of cations, such as Ca^2+^, Mg^2+^, and Cu^2+^, might grant valuable biological properties to the gels, as these metals have antibacterial properties, are capable of promoting cell proliferation and differentiation, increase growth factors expression, and stimulate angiogenesis [41,42]. Thus, these cation-mediated gels might have a promising future in wound management and tissue engineering.

## 3. Materials and Methods

### 3.1. Sample Collection and Bacterial Isolation

During different sampling campaigns, samples were collected from different sources in three marine sites in French Polynesia, southern Pacific Ocean, namely, Moorea Island lagoon (Society Archipelago) Fakarava atoll (Tuamotu Archipelago), and Tikehau atoll (Tuamotu Archipelago) (Table 1). In the laboratory, immediately upon retrieval, all samples were treated according to their nature. Enrichment cultures in appropriate marine medium led to a collection of up to 2000 isolates. Around 10% of the bacterial collection was screened for the ability to secrete EPS. The isolates were plated on 2216E (Difco™, Sparks, MD, USA) [43,44] solid medium supplemented with glucose (Interchem, Auckland, new Zealand) at a concentration of 30 g L^−1^ and incubated at ambient temperature. The bacteria were selected based on their ability to show a mucoid phenotype (Figure 1). Six isolates belonging to *Alteromonas* genus were used in this study (Table 1). The strains were deposited at Collection Nationale de Cultures de Microorganismes (CNCM) of Pasteur Institute under the Budapest Treaty.

### 3.2. EPS Production and Extraction

EPS production was performed at 30 °C in a 1-liter fermenter (New Brunswick, Toulouse, France), containing 1 L of 2216 E-glucose broth. A batch of the culture medium was inoculated at 10% (*v/v*) with a suspension of cells in exponential growth phase. The pH was adjusted and maintained at 7.6 by automatic addition of NaOH (Tikitea, Tahiti, French Polynesia) 2 mol L^−1^. Foaming was avoided by addition of Pluronic-PE6100 oil (PMC Ouvrie, Carvin, France), and the agitation rate was controlled at 200–1200 rpm to maintain the dissolved oxygen concentration at 40% of the air saturation. Glucose supplementation was 6% (60 g L^−1^) for all strains. Fermentations were stopped when glucose was consumed, between 24 up to 48 h, according to the strain [45,46]. Water-soluble exopolysaccharides were recovered from the culture medium by high-speed centrifugation (20,000× *g* for 2 h, at 25 °C), then purified by ultrafiltration against deionized water using a pellicon-2 Mini Holder equipped with a Biomax 100 K filter (Millipore Corporation, Bedford, MA, USA). Supernatants were filtered on Poly Ether Sulfone (PES) before ultimate concentration and lyophilized prior to further analysis.

### 3.3. EPS Characterization

#### 3.3.1. EPS Composition

The total neutral carbohydrate and uronic acid contents were determined by the method proposed by orcinol-sulfuric method [47,48] and the meta-hydroxydiphenyl method [49], respectively. The molar ratio of monosaccharides was determined according to Kamerling et al. [50] and Montreuil et al. [51]. The monosaccharides were analyzed after either aqueous hydrolysis or acidic methanolysis of the polymers and subsequent GC analyses as peracetylated derivatives or trimethylsilyl derivatives, respectively. Each value is a mean of three determinations.

Protein content was quantified by a modified Lowry method, as described by Concórdio-Reis et al. [52]. For the determination of inorganic content, lyophilized EPS samples (~40 mg) were placed at 100 °C until a constant weight was attained. The dried EPS samples were subjected to pyrolysis (550 °C, 24 h), and weighted for the gravimetric quantification of the inorganic content. Acyl groups were analyzed by HPLC with an Aminex HPX-87H 300 × 7.8 mm column (Biorad, Hercules, CA, USA) coupled with UV detector (210 nm), as described by Concórdio-Reis et al. [52]. Sulfate concentration in the hydrolysates was determined by HPLC using a Thermo Ionpac AS9-HC 250 × 4 mm column and a Thermo Ionpac AG11HC column (Thermo Scientific™ Dionex™, Sunnyvale, CA, USA), equipped with a conductivity detector. The analysis was performed at 25 °C, using sodium acetate (8 mM) at a flow rate of 1 mL min^−1^. All analysis were performed in duplicate.

#### 3.3.2. Fourier Transform Infra-Red (FTIR) Spectroscopy

FTIR were recorded on a Spectrum II spectrometer (Perkin-Elmer, Llantrisant, UK). The spectra were obtained between 500 and 4500 cm^−1^ after 10 scans, at room temperature.

#### 3.3.3. Molecular Mass Distribution

The analyses were performed on a Prominence HPLC system from Shimadzu equipped with a flow refractive index detector (RID20A from Shimadzu, Kyoto, Japan), a UV detector (SPD40A from Shimadzu operating at 280 nm) and a DAWN HELEOS Light Scattering detector from Wyatt, operating at 18 scattering angles and at a wavelength of 660 nm. Separation was performed with a guard column SHODEX OH PACK SB-G-6B and two SHODEX columns OH PACK SB-806MHQ (13 µm, 300 × 8 mm) and SB-805 HQ (7 µm, 300 × 8 mm). The average molar masses (number average molar mass Mn and weight average molar mass Mw) and the dispersity index (Ð = Mw/Mn) were derived both from the RI and MALS signal using a specific refractive index increment (dn/dC) of 0.135 mL/g. 

#### 3.3.4. Thermogravimetry (TG) Analysis

EPS samples (~10 mg) were characterized by Thermogravimetry (TG) using a Thermogravimetric Analyzer Labsys EVO (Setaram, Caluire, France), with a heating rate of 10 °C·min^−1^, from 25 to 500 °C.

### 3.4. EPS Functional Properties

#### 3.4.1. Rheological Properties

The apparent viscosity of the EPS aqueous solutions (1 *w/v*%) was measured at 25 °C using a controlled stress rheometer (Haake Mars III–Thermo Scientific, Karlsruhe, Germany), with a UTC–Peltier system to control temperature, and a cone-plate sensor system (angle 2°, diameter 35 mm). Measurements were carried out using a stationary shear flow, according to an adapted method from Freitas et al. [37]. 

#### 3.4.2. Gel Forming Capacity

The gel forming capacity of the EPS was tested by preparation of cation-mediated gels using divalent cations (FeSO_4_·7H_2_O, Honeywell Fluka, Seelze, Germany, CuSO_4_·5H_2_O, AppliChem Panreac, Barcelona, Spain, CaCl_2_·2H_2_O, AppliChem Panreac, Barcelona, Spain, and MgSO_4_·7H_2_O, BioChem Chemopharma, Cosne sur Loire, France) and a trivalent cation (FeCl_3_·6H_2_O, Alfa Aesar, ThermoFisher, Kandel, Germany). The gelation studies were performed according to the procedure described by Shimada et al. [53], with minor modifications: 5 mL of EPS solution (5 g L^−1^) was added to the metal salt (10 mg of cation) and agitated until dissolution and gel formation was assessed (standard conditions). Afterwards, 1 mL of NaOH (2 M) was added and the solution was agitated to test gelation in alkaline conditions. Gel formation was assessed by visual inspection, and the gels were categorized according to their strength and homogeneity: (+) for homogeneous gels that maintained their gel structure in a tube-inversion test, (-) for homogeneous gels that did not sustain their structure in the inversion test, and (--) for small non-homogeneous gels.

## 4. Conclusions

The exopolysaccharides from six *Alteromonas* strains collected from French Polynesia marine environments were characterized and investigated for their functional properties. The *Alteromonas* strains produced high molecular weight heteropolysaccharides, with high contents in uronic acids and rare sugars in their composition. These monomers, together with the sulfate groups also detected in their composition, indicate that these EPS could have interesting biological properties that are worthy to explore. Moreover, the EPS showed a good thermal stability, interesting rheological properties, and gel forming capacity, envisaging their potential to be used as structuring or thickening agents in wound management, tissue engineering, drug delivery, food products, cosmetic formulations, and oil drilling fluids.

## Figures and Tables

**Table 1:** Isolates’ identification, sampling location and sample source of the EPS producing *Alteromonas* strains isolated from marine environments in French Polynesia.

EPS	Isolate Strain Designation	Sampling Site	Sample Source	Accession Number
A	Mo 169	Moorea Island lagoon	Giant clam (*Tridacna maxima*)	CNCM I-5374
B	Mo 278	Moorea Island lagoon	Sea Anemone (*Actiniaria*)	CNCM I-5375
C	Fak 1576	Fakarava atoll	Coral	CNCM I-5376
D	Tik 650	Tikehau atoll	“Kopara” mat	CNCM I-5523
E	Fak 1386	Fakarava atoll	Pearl oyster mantle	CNCM I-5524
F	Mo 203	Moorea Island lagoon	“Kopara” mat	CNCM I-3970

**Table 2:** Characterization of EPS produced by Alteromonas strains isolated from French Polynesia: monosaccharide (Fuc, fucose; Gal, galactose; GalA, galacturonic acid; Glc, glucose; GluA, glucuronic acid; Man, mannose; and Rha, rhamnose) and acyl groups (Ac, acetate; Pyr, pyruvate; and Suc, succinate) composition; sulfate, uronic acids, protein, and inorganic salts content; average molecular weight (Mw); polydispersity index (PDI); and thermal degradation temperature (Tdeg).

EPS	Monosaccharide Composition (Molar Ratio)	Acyl Groups (wt%)	Sulfate (wt%)	Protein (wt%)	Inorganic Content (wt%)	M_w_ (MDa)	PDI	T_deg_ (°C)
A	Glc:GlcA:Man:Gal:GalA (2.5:2.5:1.5:1.5:1.5)	Ac (0.5 ± 0.1) Pyr (4.9 ± 0.1)	2.8 ± 0.1	2.8 ± 0.4	7.7 ± 1.5	1.6 4.6 ^1^	1.3 1.3 ^1^	269
B	Gal:GlcA:Glc:GalA (2:1.5:1:1)	Ac (0.5 ± 0.0) Pyr (0.2 ± 0.0) Suc (1.8 ± 0.1)	3.3 ± 0.3	3.5 ± 0.1	20.0 ± 1.7	4.6	1.4	265
C	Gal:Rha:Man:Glc:GlcA:GalA:Fuc (4.5:2:1.5:1:1:1:0.5)	Pyr (1.1 ± 0.2)	3.4 ±0.0	2.6 ± 1.0	34.7 ± 0.2	1.2	1.4	268
D	GlcA:Glc:Gal:Man:GalA (3:2:2:1.5:1.5)	Ac (2.1 ± 0.0)	2.0 ± 0.0	6.8 ± 0.2	11.4 ± 0.3	1.4	1.5	262
E	Gal:Man:Rha:Glc:GlcA:GalA (3:2:1:1:1:1)	Suc (1.0 ± 0.0)	2.9 ± 0.0	2.1 ± 0.3	15.0 ± 0.1	2.5 4.3 ^1^	1.1 1.5 ^1^	267
F	Glc:Gal:GlcA:Rha:GalA (2:2:2:1:1)	Ac (0.7 ± 0.0) Pyr (5.5 ± 0.3)	3.4 ± 0.0	1.7 ± 0.0	13.7 ± 0.3	3.2	1.2	260

^1^ EPS presented two distinct peaks in the size exclusion chromatography plots.

**Table 3:** Carreau model parameters estimated for different aqueous solutions (1% *w*/*w*) prepared with EPS produced by *Alteromonas* strains isolated from French Polynesia.

EPS	*η*_0_ (Pa.s)	*n* (−)	λ (s)	r^2^	MRE * (%)
A	76.2 ± 2.1	0.181 ± 0.051	24.6 ± 2.8	0.999	13.6
B	16.1 ± 0.3	0.361 ± 0.024	19.7 ± 1.5	0.999	9.8
C	11.3 ± 0.2	0.437 ± 0.021	17.7 ± 1.5	0.999	14.9
D	0.74 ± 0.02	0.549 ± 0.034	2.26 ± 0.27	0.998	5.6
E	26.3 ± 0.4	0.338 ± 0.025	21.2 ± 1.6	0.999	5.9
F	0.09 ± 0.01	0.772 ± 0.056	0.07 ± 0.04	0.998	3.1

* MRE=∑i=1j|(xexpi−xmodeli)xexpi|/j×100.

**Table 4:** Gel formation under standard and alkaline conditions was assessed according to their strength and homogeneity: (+) for homogeneous gels that maintained their gel structure in an inversion test, (-) for homogeneous gels that did not sustain their structure in the inversion test, (--) for small non-homogeneous gels, and blank for no gelation observed.

Standard Conditions						
**Cation**	**A**	**B**	**C**	**D**	**E**	**F**
Mg^2+^						
Cu^2+^	-		-		--	
Ca^2+^						
Fe^2+^			+		+	
Fe^3+^	-				--	-
**Alkaline conditions**						
**Cation**	**A**	**B**	**C**	**D**	**E**	**F**
Mg^2+^	+	-	-	--	-	+
Cu^2+^		+	--		--	
Ca^2+^	+		-	--	--	+
Fe^2+^	-	-	--	--	--	-
Fe^3+^	--		--	--	--	

## Data Availability

The data presented in this study are available on request from the corresponding author. The data are not publicly available due as public availability violates the consent given by the study participants.

## Appendix A

Examples of the two distinctive HPSEC results, one and two chromatographic peaks, obtained from molecular mass distribution determination (Figure A1).

## Appendix B

Thermal degradation steps of the EPS (Table A1).

**Table A1:** Thermal degradation steps of the EPS produced by *Alteromonas* strains isolated from French Polynesia.

EPS	Temperature (°C)	Weight Loss (%)
A	40.20–192.81 193.18–405.15	14.6 42.5
B	40.59–186.14 186.33–418.89	13.8 36.4
C	37.97–189.07 189.47–428.81	14.2 30.2
D	38.6–183.88 184.46–385.68	12.7 45.1
E	36.61–188.77 189.49–390.41	17.5 44.8
F	45.14–73.09 212.40–385.88	12.1 41.5

## Appendix C

Images of gels homogeneous gels that did not sustain their structure in the inversion test (-), and small non-homogeneous gels (--) (Figure A2).
